# Ion-Abrasion Scanning Electron Microscopy Reveals Surface-Connected Tubular Conduits in HIV-Infected Macrophages

**DOI:** 10.1371/journal.ppat.1000591

**Published:** 2009-09-25

**Authors:** Adam E. Bennett, Kedar Narayan, Dan Shi, Lisa M. Hartnell, Karine Gousset, Haifeng He, Bradley C. Lowekamp, Terry S. Yoo, Donald Bliss, Eric O. Freed, Sriram Subramaniam

**Affiliations:** 1 Laboratory of Cell Biology, Center for Cancer Research, NCI, NIH, Bethesda, Maryland, United States of America; 2 HIV Drug Resistance Program, NCI, Frederick, Maryland, United States of America; 3 FEI Company, Hillsboro, Oregon, United States of America; 4 National Library of Medicine, NIH, Bethesda, Maryland, United States of America; Northwestern University, United States of America

## Abstract

HIV-1-containing internal compartments are readily detected in images of thin sections from infected cells using conventional transmission electron microscopy, but the origin, connectivity, and 3D distribution of these compartments has remained controversial. Here, we report the 3D distribution of viruses in HIV-1-infected primary human macrophages using cryo-electron tomography and ion-abrasion scanning electron microscopy (IA-SEM), a recently developed approach for nanoscale 3D imaging of whole cells. Using IA-SEM, we show the presence of an extensive network of HIV-1-containing tubular compartments in infected macrophages, with diameters of ∼150–200 nm, and lengths of up to ∼5 µm that extend to the cell surface from vesicular compartments that contain assembling HIV-1 virions. These types of surface-connected tubular compartments are not observed in T cells infected with the 29/31 KE Gag-matrix mutant where the virus is targeted to multi-vesicular bodies and released into the extracellular medium. IA-SEM imaging also allows visualization of large sheet-like structures that extend outward from the surfaces of macrophages, which may bend and fold back to allow continual creation of viral compartments and virion-lined channels. This potential mechanism for efficient virus trafficking between the cell surface and interior may represent a subversion of pre-existing vesicular machinery for antigen capture, processing, sequestration, and presentation.

## Author Summary

Current treatment regimens for HIV-infected individuals are not capable of eradicating HIV infection, even though combinations of highly potent antiviral drugs are used. Indeed, drug regimens must be periodically altered as the virus resurges from a persistent reservoir. Macrophages, which serve as “search-and-destroy” immune surveillance cells of the body, are now thought to be a key component of this reservoir. Evidence suggests that macrophages can harbor infectious HIV virions for long periods, and transmit them to bystander T cells. We have used a new technique called ion abrasion scanning electron microscopy (IA-SEM) to image entire HIV-infected human macrophages at a resolution high enough to see individual HIV virions and their location within the cell. This approach revealed that HIV is present in a system of nanoscale tubes, barely larger than a virus at some places, which connect internal viral reservoirs to the cell surface. These tubes could allow the macrophage to deliver HIV virions to bystander cells from its continually replenished stores of ammunition, held deep within the cell. Our work provides a glimpse of how the structure of these reservoirs allows macrophages to accomplish viral delivery. Discovery of these virion-channeling tubes provides a potential drug target to address the problem of persistent HIV infection.

## Introduction

Conventional transmission electron microscopy is a powerful tool for investigation of HIV pathogenesis and subcellular organization of viral compartments in infected cells [1]. Insights into cellular ultrastructure using this approach have generally been obtained using thin sections derived from fixed, plastic-embedded cells or tissues. However, the use of sectioned material provides an incomplete representation of cellular architecture, since a 200 nm section typically includes <1% of the volume of the complex mammalian cell. Thus, a compartment that appears to be completely internal in one section might be revealed as an invagination of the cell membrane in another section, while what appears to be a vesicle in one section might turn out to be a tubular membrane process in another. Because of the incompleteness of data obtained from cell sections, the probability of visualizing a full and accurate representation of the cytoarchitecture is minimal. This limitation is overcome with the use of ion abrasion scanning electron microscopy (IA-SEM), a newly developed approach for 3D imaging of large mammalian cells and tissues in their entirety [2],[3],[4] at in-plane spatial resolutions of ∼6 nm and z-axis resolutions of ∼30 nm.

The functional importance of obtaining cellular images that go beyond the information obtained from a thin section is illustrated by recent controversy surrounding the biogenesis of human immunodeficiency virus type I (HIV-1) in macrophages. Thus, contradictory inferences about the connectivity and surface accessibility of viral compartments have been drawn using light microscopy [5],[6] and electron microscopy of sections from cells treated with a membrane-impermeant dye [7]. Macrophages are a persistent and long-lived reservoir of provirus and infectious virions in HIV-1 infection [8],[9],[10],[11],[12]. They can survive for weeks while being productively infected, and during this time may repeatedly mediate directed, efficient infection of T cells [11],[13]. Previous reports have suggested that, in macrophages, HIV-1 virions assemble either in late endosomes [14], at the plasma membrane [6], in internal compartments that communicate with the plasma membrane and bear markers of late endosomes [7],[15], or at a combination of these sites [5]. Release of infectious virions is not affected when late endosome motility is disabled [6]; yet, internally sequestered HIV-1 is rapidly directed to newly-formed synapses with T cells [5], suggesting that movement of virions from internal compartments to the plasma membrane plays a role in directed infection. Since IA-SEM should allow determination of the location and distribution of viruses and viral compartments through the depth of the cell, we applied this method to definitively resolve the controversy about the precise localization and distribution of HIV-1 in infected macrophages.

## Materials and Methods

### Ion Abrasion Scanning Electron Microscopy

Resin blocks from standard flat embedding moulds (EMS, Hatfield, PA) were trimmed to a pyramidal shape using a razor blade with block faces typically of about 2 mm^2^ in area. The surface was smoothened by sectioning using a conventional 45° diamond knife from Diatome (distributed by EMS, Hatfield, PA). The entire pyramidal block was removed and mounted with the wider base onto an SEM stub using silver paint (SPI Supplies, West Chester, PA) such that the ultramicrotome-prepared, flat surface of the resin block pointed upwards, perpendicular to the electron column. For room temperature experiments, images were recorded using a Nova 200 NanoLab dual beam instrument (FEI, Hillsboro, OR) equipped with a gallium ion source for focused ion beam milling and a field emission gun scanning electron microscope with an in-lens secondary electron detector for imaging. Prior to milling and SEM imaging, the entire sample surface was coated with a platinum/palladium layer (∼1 µm thickness) using the gas injector system (GIS) in the main specimen chamber. The specimen stage was tilted to 52° and exposed to the focused ion beam (such that the plane of the stage was parallel to the ion beam). A cross sectional cut was introduced in two stages. First, a coarse cut was made at high beam currents (typically 7–20 nA) and at an accelerating voltage of 30 kV to create a trench that enabled viewing of the cross-section. Usually, 50–150-µm-wide trenches were cut into the specimen. In the second step, the ion beam was scanned using a current of 3–7 nA to polish and smoothen the surface. Secondary electron SEM images were typically recorded at accelerating voltages of 3 kV, 10,000× magnification, and a beam current of 68–270 pA in the immersion lens mode. For slice-and-view images series, a step size of ∼15 nm was chosen for the removal of material from the specimen surface using the focused ion beam. All images are presented with inverted contrast in the figures and videos for ease of comparison to images obtained from transmission electron microscopy.

### Preparation of HIV-1-Infected Macrophages and T Cells

Primary monocyte-derived macrophages were infected with either (i) vesicular stomatitis virus G glycoprotein (VSV-G)-pseudotyped, Env-defective HIV-1 virus stocks produced by co-transfection of 293T cells with pNL4-3/KFS/MA-TC [5],[16] and the VSV-G expression vector pHCMV-G [17] or (ii) infectious HIV-1 BaL. We used Env-defective HIV-1 viruses for most of the studies to eliminate the possible formation of an apparently internal compartment resulting from fusion of two previously separate cells. The electron tomographic analyses with fixed, embedded cells were carried out with infectious HIV-1 BaL. Three days post-infection, the infected macrophages were harvested and processed for electron tomographic and IA-SEM experiments. Jurkat T cells were cultured in RPMI-1640 medium supplemented with 10% FBS and infected for 4–5 hrs with NL4-3/29/31KE virions pseudotyped with VSV-G. Infected cells were harvested 24–48 hrs post-infection and processed for electron microscopy.

### Preparation of HIV-Expressing Macrophages for IA-SEM

HIV-1-expressing macrophages were prepared for imaging by adding freshly prepared 2X fixative buffer (5% glutaraldehyde in 0.2 M sodium cacodylate) to the cell culture medium in a 1∶1 ratio immediately after pre-warming it to the cell culture temperature, followed by incubation at this temperature for 30 min. The 2X fixative buffer was then replaced by fresh 1X fixative buffer (2.5% glutaraldehyde, 0.1 M sodium cacodylate), followed by incubation at room temperature for 15 min. The cells were centrifuged at 12,000×g and the pellet trimmed into small blocks (∼2 mm square) with an acetone-cleaned razor blade. The samples were transferred into glass scintillation vials containing 0.1 M sodium cacodylate buffer, rinsed 3 times with the buffer for 10 minutes each, and post-fixed with 1% osmium tetroxide in 0.1 M sodium cacodylate buffer (OsO_4_) for 1 hr, followed by 2 more washes in 0.1 M cacodylate buffer for 10 minutes each. The samples were washed in a cold sodium acetate buffer (0.1 M) once and stained *en bloc* with 0.5% uranyl acetate in 0.1 M acetate buffer for 1 hr, followed by 3 more washes in acetate buffer. The samples were dehydrated through graded ethyl alcohol followed by propylene oxide. Samples were then infiltrated overnight at room temperature with a 1∶1 mixture of Epoxy resin: propylene oxide, embedded in Embed 812 (Electron Microscopy Sciences, Inc.) and cured for 48 hrs in a 55°C oven.

### Cryo-Electron Tomography of HIV-1 Infected Macrophages

Monocyte-derived macrophages (MDM) from healthy donors were grown in RPMI-1640 media supplemented with 10% fetal calf serum. The cells were plated on gold Quantifoil grids (Quantifoil Micro Tools GmbH, Germany) and infected with VSV-G pseudotyped HIV-NL4-3/MA-TC virus for 4 hrs (10^6^ cells+10^5^ RT cpm virus). The cells were washed gently and incubated in media at 37°C for a further 4 days, with a change in culture medium after 2 days. They were then fixed overnight in 2.5% glutaraldehyde and rinsed with PBS. After deposition of 15 nm-sized gold fiducials on the grid, the cells were rapidly frozen by plunging the grid into liquid ethane maintained at ∼−180°C using a Vitrobot device (FEI Company, Oregon). The grids were imaged at liquid nitrogen temperatures on a Titan Krios electron microscope (FEI Company, Oregon) equipped with a Gatan 2002 energy filter and operated at 200 kV. Low dose tomographic tilt series were collected over a tilt range spanning ±65° in 1.5° intervals with a total dose of ∼75 e^−^/Å^2^, with an applied defocus of −15 µm, and an effective pixel size of 1.9 nm at the specimen plane. Tomograms were reconstructed using the software package IMOD [18],[19].

### Electron Tomography of Thin Sections

MDM that had been infected for 7 days with the primary isolate strain HIV-1 BaL were provided by Tracy Hartman and Robert Buckheit (Imquest Biosciences, Frederick, MD), and prepared for sectioning with the same procedure used for IA-SEM above. Samples were cut into 90–100 nm-thick sections using a Leica Ultracut T Microtome (Leica Microsystems, Vienna, Austria), placed on carbon-coated copper EM grids, stained with lead citrate, coated with 10 nm gold beads (Nanoprobes, Yaphank, NY), and imaged in a Tecnai 12 transmission electron microscope at 120 kV, 52,000× magnification (image pixel size = 0.44 nm), and applied defocus of −1 µm. Tilt series were recorded using the Xplore3D software (FEI, Netherlands), and the Saxton tilt scheme with an initial tilt increment of 2°. Data were collected either as single axis tilt series and reconstructed using weighted back-projection (for fixed, embedded sections), or as dual axis tilt series which were aligned, reconstructed, and merged into dual-axis tomograms (data in Video S1). Reconstructions were carried out in the environment of the software package IMOD [18],[19].

### 3D Visualization of IA-SEM Image Stacks

Image stacks were aligned using the Inspect3D software package (FEI, Netherlands). Individual virons were automatically identified with a spherical Hough transform. Segmentation of the cell was accomplished with a combination of region growing and level-set methods found in the Insight Toolkit (http://www.itk.org). These results were then verified and refined with manual classification, specifically for the details in the virion channels, with Slicer3D (http://www.slicer.org). The resulting models were then rendered with 3ds MAX software using Brazil, a rendering plug-in.

## Results

**Figure 1:**
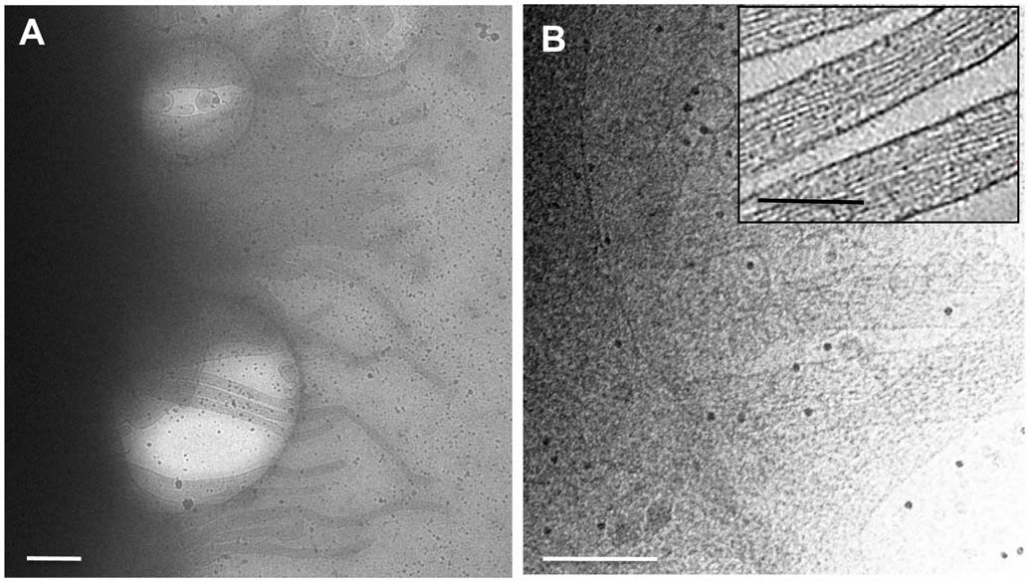
Cryo-electron tomography of HIV-1 infected monocyte-derived macrophages (MDM). (A) Projection image of the edge of an infected cell at low magnifications indicating that no useful image contrast is obtained in most regions of the cell except at the very outer edges. (B) Projection image at higher magnification near the edge of the cell showing filopodial extensions and clusters of virions in close proximity. The inset shows a magnified view of individual filopodia, denoised to enhance visualization of the actin bundles that make up the interior. The small black dots that are especially noticeable in panel (B) represent 15 nm-sized gold particles added to the grid for purposes of providing fiducials for tomographic reconstruction. Scale bars: panel (A) 1 µm, panel (B) and inset 200 nm.

**Figure 2:**
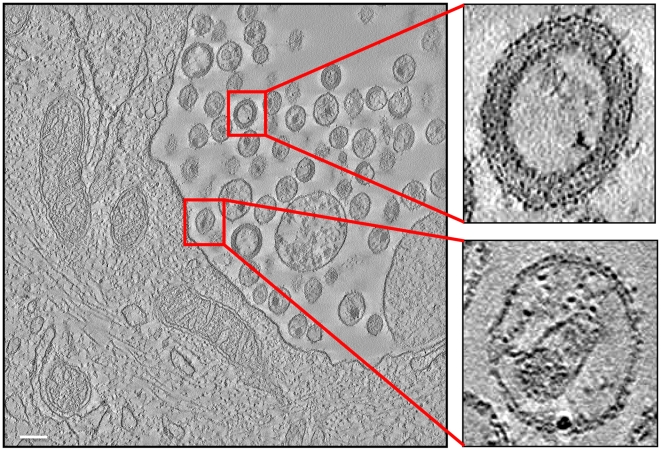
Electron tomography of a 150 nm thick section from HIV-1 BaL infected macrophages. A tomographic slice (nominal thickness 1 nm) through a region of the cell containing a collection of viruses in an internal compartment. The expanded insets show zoomed-in images of individual immature (top) and mature (bottom) virions. Scale bar 100 nm.

We first present cryo-electron tomographic studies of primary MDM infected with HIV-1. Projection images of whole cells on Quantifoil grids show that useful images can only be recorded at the very outer edges of the cells (Figure 1A), where long filopodial extensions can be seen to emanate from the cell surface (Figure 1B). Tomograms recorded from these filopodial extensions show the remarkable proximity and association of released virions to the actin-rich filopodial structures (Figure 1B) even at regions many microns away from the main body of the cell. Complementary information on subcellular architecture was obtained from electron tomography of thin sections from fixed, plastic-embedded HIV-1 infected macrophages, which provides a view of the interior of the cell (Figure 2 and Video S1). Inspection of the tomographic data from the interior of the cell shows clear examples of budding, immature and mature virions inside the compartment, but does not provide the spatial connections, if any, between these internal compartments and the surface of the cell.

**Figure 3:**
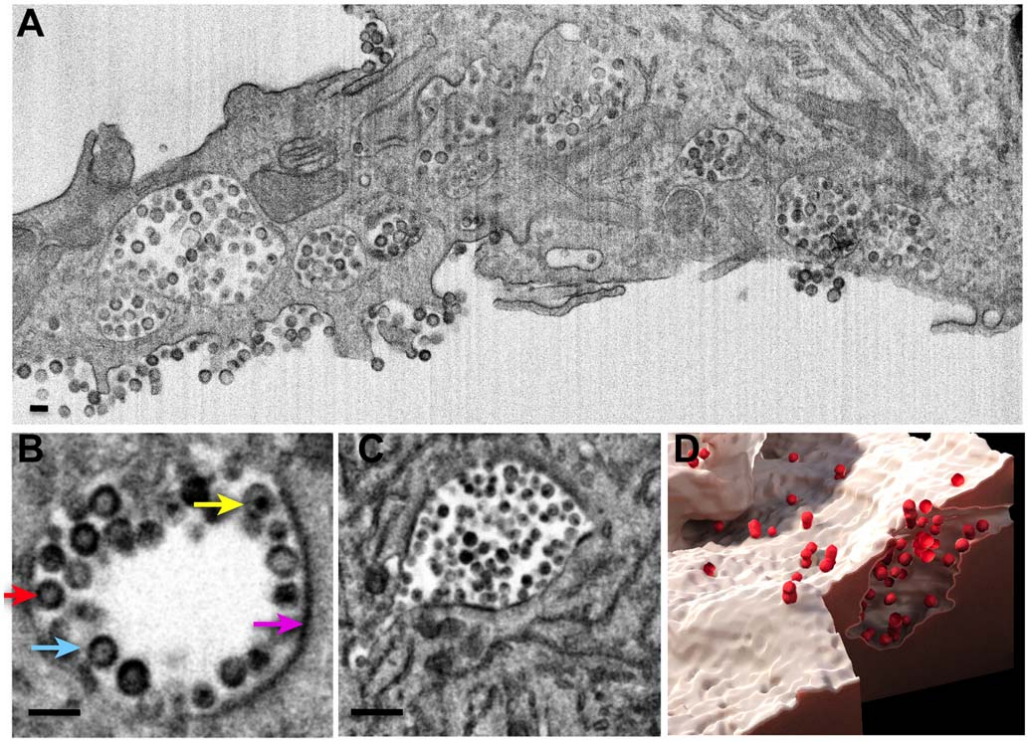
Virion reservoirs and channels in primary HIV-1-infected MDM revealed by IA-SEM imaging. (A) Single cross-sectional image shows internal compartments, highlighted further in (B) indicating budding (light blue arrow), immature (red arrow), and mature (yellow arrow) virions, and membrane boundary (pink arrow). (C) Some compartments deep in the interior are seen to display “bagpipe”-like protrusions that are filled with virions. Scale bars are 200 nm long in panels (A)–(C). (D) Segmented 3D image illustrating the distribution of virions (red) within a reservoir connected to the plasma membrane. A 2D scale bar cannot be used for the 3D perspective rendering in (D); for reference, however, each virion has an approximate diameter of 120 nm.

**Figure 4:**
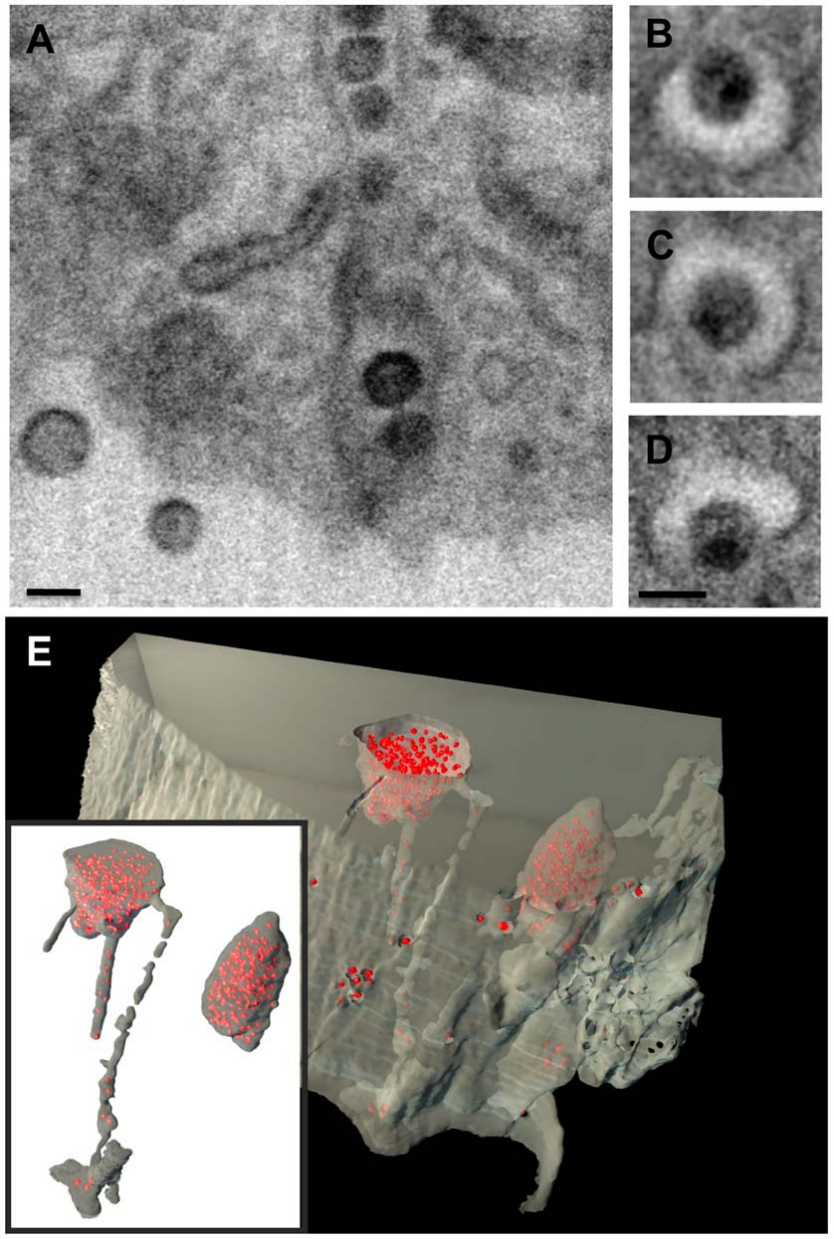
Virion channels of roughly uniform diameter allow communication between deep internal reservoirs and the plasma membrane of primary HIV-1-infected MDM. Individual IA-SEM images of virion channels identified by dual beam imaging are shown in (A) transverse and (B–D) axial sections. Scale bars are 100 nm long. (E) and inset: Illustration of the depth of some of the virion channels by automated segmentation of the raw 3D image (shown in Video S2) of a portion of an HIV-1 infected macrophage with two internal compartments, each of which contains numerous virions (red) with an approximate diameter of ∼120 nm. Virion channels connecting to the surface are observed from the compartment on the left, while the compartment on the right is completely internal. An animation of the segmented volume is included in Video S7.

Since cryo-electron tomography of intact cells is limited to visualizing the thin edges of the cell, and conventional electron tomography is limited to imaging thin sections, we used IA-SEM to carry out 3D imaging of the infected macrophages through large segments of the central, thicker regions of the cell (Videos S2, S3, S4, S5, S6). We used Env-defective HIV-1 virions for these experiments in order to eliminate the possible formation of an apparently internal compartment resulting from fusion of two previously separate cells. Inspection of a 3D image stack shows that regions from which viruses are released at the plasma membrane are connected to larger viral compartments deeper in the cell (Figures 3 and 4, Video S3). These internal compartments contain immature, mature, and budding HIV-1 virions (Figure 3B), establishing that they are sites of virogenesis. Sites of viral budding were also observed at the cell surface associated with electron-dense plasma membrane patches (Video S5), as were immature and mature virions, confirming that HIV-1 is not only assembled in internal compartments in primary MDM, but can be simultaneously assembled at the plasma membrane.

Surprisingly, IA-SEM imaging revealed highly structured tubules in the cytoplasm (these can be seen in Figure 4, but are especially clear in Video S6, in which a long HIV-1 containing tubular structure can be visualized starting from the top portion of the stack). These tubules, which have a roughly uniform diameter of ∼150–200 nm, and lengths of up to 5 µm, project from the virion-containing compartments, occasionally connecting them to the cell surface (Figure 4, Supplementary legends, Videos S2, S3, S6). The presence of a chain of HIV-1 virions within these tubular compartments suggests that they permit movement of virions between the cell surface and the intracellular compartments, and we therefore refer to them as “virion channels”. These tubular structures differ markedly from the narrow (<20 nm), opposed macrophage membrane sheets reported previously [15] which were not associated with virions.

**Figure 5:**
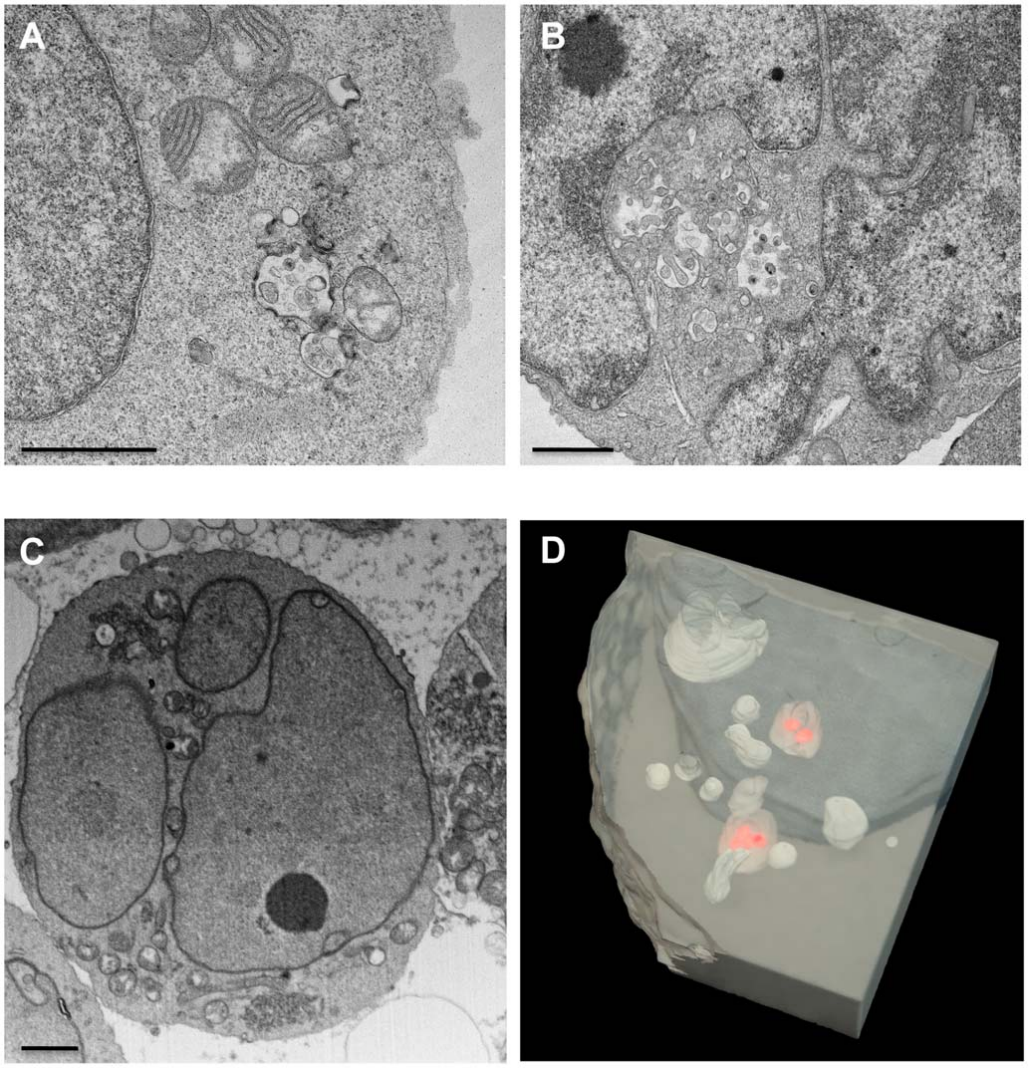
Transmission electron microscopic and IA-SEM imaging of Jurkat T cells infected with the VSV-G pseudotyped 29/31 HIV-1 Gag matrix mutant. (A, B) Selected projection TEM images from a 100 nm thick section obtained from fixed, osmium-stained, plastic-embedded cells. Small vacuolar compartments, some containing viruses can be visualized in the interior of the cell. (C) A single IA-SEM image from the interior of the same block used to obtain the TEM images in panel (A). A complete stack of images showing the distribution and closed shapes of the compartments through this and another cell is included in Videos S8 and S9. (D) Rendering of the membrane conpartments in the interior of the cell illustrating that they are closed, and not connected to the cell surface irrespective of whether or not they appear to contain viruses (red). Scale bars in all panels are 1 µm wide.

Some of the deeper compartments well inside the interior of the cell do not display detectable cell surface communication (Figure 4E, Video S4, with animation in Video S7), potentially representing multivesicular body compartments. Since the studies we report here use cells fixed with glutaraldehyde, one possible concern is that the use of the fixative may alter the shapes of internal compartments because of local changes in osmolarity in the fixation medium. Previous electron tomographic studies of fixed, stained B-cells have suggested that the use of glutaraldehyde did not alter the overall shape of lysosomes, but led to some shrinkage and deformation of early and late endosomes [20]. Although it is difficult to imagine how an entire system of virion channels with connections to the cell surface can be created instantaneously by local deformations, we evaluated this possibility by carrying out control experiments with Jurkat T cells, taking advantage of the recent demonstration by Joshi et al [21] that the HIV-1 29/31 KE Gag matrix mutant is targeted to multivesicular bodies in Jurkat T cells. Both transmission electron microscopic (Figure 5A) as well as IA-SEM (Figure 5B, Video S8, S9) analyses reveal that the MVB compartments in these cells are closed and display no evidence of virion channels that are connected to the cell surface. These experiments show that the virion channels are a specialized feature of macrophages and possibly other antigen-presenting cells, not present in all HIV-infected cell types, and unlikely to be an artifact of glutaraldehyde fixation.

**Figure 6:**
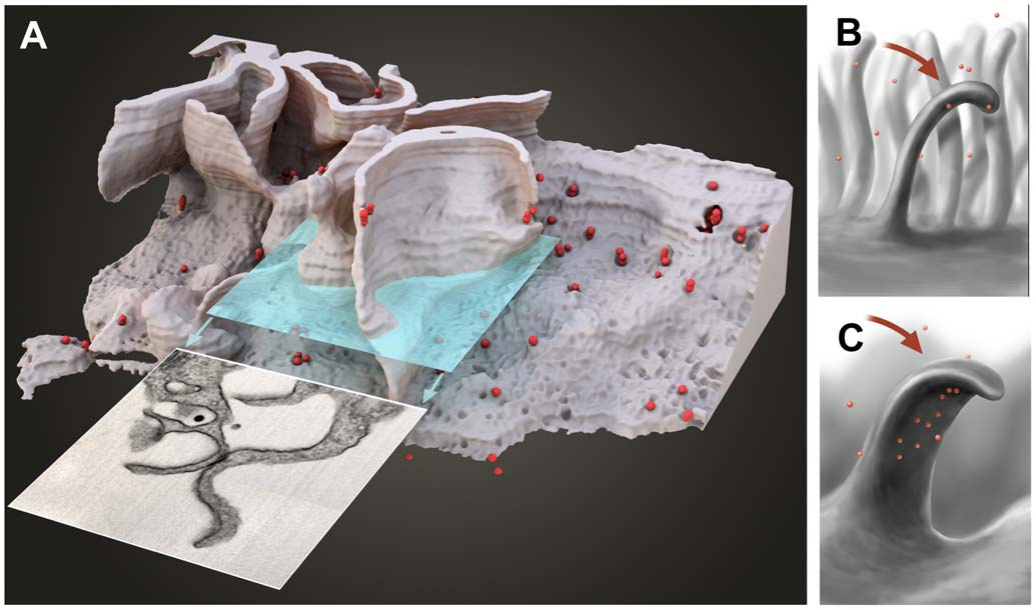
3D representation of the surface and interior of an HIV-infected macrophage (animation is presented in Video S10). (A) Sections that would appear to contain “filopodia” when imaged by transmission electron microscopy of individual sections can actually correspond to large wavelike membrane processes as in this example. The virions are shown in red. (B, C) Schematic side (B) and front (C) views of these surface protrusions shown to indicate how bending and folding back of the extensions onto the surface of the cell could trap the contents of the aqueous environment within the invaginated folds of the membrane, and allow creation of viral compartments.

IA-SEM studies also reveal new insights into the membrane organization on the surfaces of macrophages, which can have extended membranous features (Figure 6A; Video S10). 3D imaging through these regions shows that some membrane processes that might be identified as “filopodia” in cross section (using transmission electron microscopy of thin sections) actually correspond to massive wavelike projections that would be far more efficient at particle capture than filopodia (Figures 6B, 6C). These membrane extensions likely correspond to the “ruffles” or “veils” previously observed by SEM imaging of the MDM cell surface [22],[23],[24],[25].

## Discussion

Serial sectioning is a method that has been used for decades to attempt 3D reconstruction by manually combining individual 2D images. This is a tedious approach even when it does work, because it requires identification of the same cell in each successive serial section, and it also only works when there is no distortion in any of the sections at the location of the particular cell of interest. In a few rare instances, when the same cell can be located in a series of sections it is possible to attempt tomography of each section, but it still does not solve the problem of material that is lost between successive sections. The use of IA-SEM circumvents these problems, and as demonstrated in this work and in recent publications from our laboratory, allows site-specific 3D reconstruction of cells and tissues at resolutions of ∼30 nm in the z-direction and 3–6 nm in the x-y plane.

Simultaneous observation of concentrated budding sites in completely internal compartments, compartments that communicate with the plasma membrane, and the plasma membrane itself is consistent with a model wherein virogenesis can be initiated at any of these sites. The observation of structured virion channels provides an explanation for productive virion release in the absence of late endosome motility that does not necessarily invoke plasma-membrane-only budding [6]. More importantly, this virion channeling system may imply a mechanism behind the efficient, directed transmission of HIV-1 from macrophages to uninfected cells [5]. This hypothetical mechanism would allow HIV-1 to remain sequestered until such contact. The virion channeling system would also allow directed release of virions without gross movement of entire endocytic compartments. Furthermore, inherent to the channeling system is the possibility that virions from a single endocytic compartment could be simultaneously directed to multiple cells. Since the channeling model does not invoke fusion of the endocytic compartment membrane with the plasma membrane, jettison of the entire compartment of stable [11] infectious virions need not occur for each synapse. The virion channeling system described here thus suggests how controlled sequestration and directed propagation of HIV-1 from infected macrophage reservoirs may be achieved.

Tubulovesicular membrane systems have been observed in numerous mammalian cell types, ranging from oxyntic cells [26], where they function to rapidly traffic secretory proteins to the cell surface, to neurons, where specific proteins involved in synaptic vesicle trafficking have been implicated in the formation of tubules similar to those reported here [27]. The reorganization of multivesicular bodies or lysosomes into tubular structures within antigen presenting cells has been reported previously, primarily in conjunction with the traffic of MHC class II and related molecules to the plasma membrane of these cells [28],[29]. It is possible that these are related to the tubular structures described in this study. It will also be important to compare the 3D structures of macrophage viral compartments with those in other antigen-presenting cells such as dendritic cells which are either infected or exposed to HIV to get a better appreciation of the generality of this phenomenon, which may be a viral subversion of an existing membrane system that normally would channel, sequester, or deliver cellular proteins or foreign antigens.

The discovery of narrow virion channels in macrophages highlights the importance of methodologies for 3D imaging of whole cells at resolutions where all cellular components can be visualized at nanometer resolution. The combination of IA-SEM and semi-automated image segmentation such as that used in this work and elsewhere [30] provide powerful tools to explore the architectures of complex eukaryotic cells to simultaneously uncover details ranging from the internal structures of viruses to the organization of compartments inside the cell.

## Supporting Information

Video S1 Slices through a dual-axis tomogram obtained from 150 nm thick cell sections cur from a fixed, stained, plastic-embedded block containing HIV-1 infected macrophages. Primary human monocyte-derived macrophages (MDM) were infected for 7 days with the primary isolate HIV-1 BaL. The region of the cell that can be explored is limited by the thickness of the section.
(9.95 MB WMV)

Video S2 Image stack obtained by ion abrasion scanning electron microscopy (IA-SEM) illustrating the interior of an HIV-1-infected MDM. The sharp straight faces in this video and in Videos S3, S4, S5 and S6 correspond to the edge of the imaging trench. Virions are visible as small particles of ∼120 nm diameter.
(9.94 MB WMV)

Video S3 Image stack obtained by IA-SEM highlighting a virion-containing vacuole (lower left corner). In this instance, the vacuole is connected to the cell surface by a virion channel that passes over it.
(10.02 MB WMV)

Video S4 Image stack obtained by IA-SEM highlighting a virion-containing vacuole. The vacuole appears to be completely internal and not connected with the cell surface. Budding, immature, and mature virions are present.
(9.83 MB WMV)

Video S5 Image stack obtained by IA-SEM highlighting a site of concentrated viral budding from an electron-dense membrane region proximal to a filopodium at the cell surface.
(4.57 MB WMV)

Video S6 Image stack obtained by IA-SEM highlighting three “virion channels” that proceed from the vacuole: one in the z-direction (first part of the stack) and two roughly in the x-y plane (visible at near the end of the stack). Budding, immature, and mature virions are present.
(9.97 MB WMV)

Video S7 Animation of segmented image of long virion channel shown in Figure 4E.
(3.09 MB WMV)

Video S8 Example of image stacks obtained by IA-SEM illustrating the interiors of Jurkat T cells infected with the 29/31 Gag matrix mutant. The cross-sectional views include the width of the entire cell, and show numerous spherical vacuoles, likely to represent endosomal compartments. There is no evidence of long virion channels in these cells.
(4.05 MB AVI)

Video S9 Example of image stacks obtained by IA-SEM illustrating the interiors of Jurkat T cells infected with the 29/31 Gag matrix mutant. The cross-sectional views include the width of the entire cell, and show numerous spherical vacuoles, likely to represent endosomal compartments. There is no evidence of long virion channels in these cells.
(4.43 MB AVI)

Video S10 View of three-dimensional segmentation of wavelike projections emanating from the plasma membrane of an HIV-1-infected MDM imaged by IA-SEM (corresponding to data presented in Figure 6A). A slice from the image stack is shown to indicate that the large wavelike surface projections could be mistakenly identified as filopodia with a “cylindrical” cross-section by conventional 2D transmission electron microscopy.
(7.21 MB WMV)
